# Transcriptional Responses of the *Bdtf1*-Deletion Mutant to the Phytoalexin Brassinin in the Necrotrophic Fungus *Alternaria brassicicola*

**DOI:** 10.3390/molecules190810717

**Published:** 2014-07-24

**Authors:** Yangrae Cho, Robin A. Ohm, Rakshit Devappa, Hyang Burm Lee, Igor V. Grigoriev, Bo Yeon Kim, Jong Seog Ahn

**Affiliations:** 1 Korea Research Institute of Bioscience and Biotechnology, Ochang, Chungbuk 363-883, Korea; E-Mail: yangraec@kribb.re.kr; 2 Joint Genome Institute, 2800 Mitchell Drive, Walnut Creek 94598, CA, USA; E-Mails: raohm@lbl.gov (R.A.O.); ivgrigoriev@lbl.gov (I.V.G.); 3 Division of Applied Bioscience and Biotechnology, College of Agriculture and Life Sciences, Chonnam National University, Buk-Gu, Gwangju 500-757, Korea; E-Mail: hblee@chonnam.ac.kr

**Keywords:** phytoalexin, brassinin, gene expression profiles, RNA-seq, necrotrophic fungus, phytoalexin detoxification, GEO Series Accession No. GSE59195

## Abstract

*Brassica* species produce the antifungal indolyl compounds brassinin and its derivatives, during microbial infection. The fungal pathogen *Alternaria brassicicola* detoxifies brassinin and possibly its derivatives. This ability is an important property for the successful infection of brassicaceous plants. Previously, we identified a transcription factor, *Bdtf1*, essential for the detoxification of brassinin and full virulence. To discover genes that encode putative brassinin-digesting enzymes, we compared gene expression profiles between a mutant strain of the transcription factor and wild-type *A. brassicicola* under two different experimental conditions. A total of 170 and 388 genes were expressed at higher levels in the mutants than the wild type during the infection of host plants and saprophytic growth in the presence of brassinin, respectively. In contrast, 93 and 560 genes were expressed, respectively, at lower levels in the mutant than the wild type under the two conditions. Fifteen of these genes were expressed at lower levels in the mutant than in the wild type under both conditions. These genes were assumed to be important for the detoxification of brassinin and included *Bdtf1* and 10 putative enzymes. This list of genes provides a resource for the discovery of enzyme-coding genes important in the chemical modification of brassinin.

## 1. Introduction

Plants protect themselves from the attack of potential pathogens. Plant resistance mechanisms include the production of antimicrobial compounds called phytoalexins. Brassinin and its derivatives are phytoalexins produced by plants of the genus *Brassica*. They are induced during the infection process by microbes, including pathogenic fungi [1,2,3]. Brassinin also has antimicrobial activity *in vitro* [4]. Mutant strains of *A. brassicicola* with cell wall integrity defects are more sensitive to brassinin [5,6]. It is possible that brassinin affects the cell integrity of pathogens similar to camalexin, a phytoalexin that disrupts the cell membrane of the bacterium *Pseudomonas syringae* [7].

Brassinin probably contributes to the resistance of plants against pathogenic fungi because of its antifungal activity [4]. In spite of the induction of brassinin, however, several fungi establish parasitic growth in *Brassica* species. Their success as parasites might be partly due to their ability to detoxify brassinin. The stem rot fungus *Sclerotinia sclerotiorum* metabolizes brassinin into its corresponding glucosyl derivatives, which have no detectable antifungal activity [8]. In comparison, the blackleg fungus *Leptosphaeria maculans* detoxifies brassinin by the unusual oxidative transformation of a dithiocarbamate to an aldehyde [9]. *Alternaria brassicicola* detoxifies brassinin by converting it into the intermediate metabolites N’-indolylmethanamine and N'-acetyl-3-indolylmethanamine [10].

We recently produced direct molecular evidence that brassinin is important in plant resistance to *A. brassicicola*. Wild-type *A. brassicicola* detoxified brassinin by transforming it into non-indolyl products during mycelial growth in glucose yeast extract broth (GYEB). The transcription factor, brassinin detoxification factor 1 (*Bdtf1*), is an important regulator of unknown enzymes that detoxify brassinin and possibly its derivatives [11]. Mutants of the *Bdtf1* gene failed to detoxify brassinin and showed a 70% reduction in virulence on *Brassica* species but no measurable effects on *Arabidopsis thaliana*, which is in the brassica family, but produces camalexin instead of brassinin. Under test conditions, wild-type *A. brassicicola* completely degraded the indolyl compound brassinin, but did not produce intermediate products, such as N'-indolylmethanamine and N’-acetyl-3-indolylmethanamine. Brassinin hydrolase in *A. brassicicola* (BHAb) produces these intermediates [12]. However, expression of the *BHAb* gene is not regulated by the transcription factor *Bdtf1* and its expression level is very low in both the ∆*bdtf1* mutant and wild-type *A. brassicicola*. The data suggest that *A. brassicicola* produces additional enzymes important for digestion of brassinin during saprophytic growth in a nutrient-rich medium containing brassinin. The aim of this study was to investigate the gene expression profiles of wild-type *A. brassicicola* and the *∆bdtf1* mutant in a search for novel genes that encode brassinin-detoxification enzymes.

## 2. Results

### 2.1. Effects of Brassinin on ∆bdtf1 Mycelium

**Figure 1:**
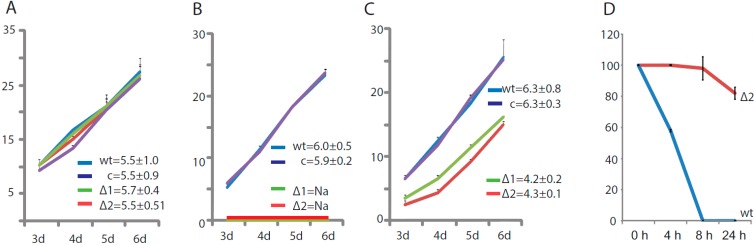
Effects of brassinin on colony growth of *∆bdtf1* mutants of *Alternaria brassicicola* on potato dextrose agar calculated as the slope of the linear regression line with four data points. Y-axes show colony diameter in millimeters and their inability to digest brassinin. (**A**). Similar growth rates in the absence of brassinin. (**B**). No growth of *∆bdtf1* mutants in the presence of 0.2 mM brassinin. (**C**). Reduced growth of *∆bdtf1* mutants in the presence of 0.1 mM brassinin. (**D**). Reduced degradation of brassinin by *∆bdtf1* during mycelial growth in a liquid medium. Y-axis shows relative amounts of brassinin compared to the input amount. Bars represent standard deviations.

Abbreviations: wt = wild-type *Alternaria brassicicola*; c = *∆bdtf1-5* mutants complemented with a native allele of the *Bdtf1* gene (*∆bdtf1:Bdtf1*); *∆1 =* (*∆bdtf1*-5); *∆2 = Bdtf1* deletion mutant (*∆bdtf1*-9).

Each strain of the ∆*bdtf1* mutants was indistinguishable from wild-type *A. brassicicola* in mycelial growth on nutrient-rich potato dextrose agar (PDA) (Figure 1A) or glucose-yeast-extract-broth medium (GYEB). They were also identical to wild-type *A. brassicicola* in conidium development and response to stress-inducing chemicals. A major difference between the mutants and the wild type was their response to brassinin [11]. Brassinin in 0.2 mM concentration caused a slight delay in germination of the wild type, but the mutants were unable to germinate. Active mycelial growth of the mutants also stopped when transferred to PDA or GYEB containing 0.2 mM brassinin (Figure 1B). In the presence of 0.1 mM brassinin the mutants germinated and grew, but their growth rate was significantly reduced (*p* < 0.01) compared to wild-type *A. brassicicola* (Figure 1C). In GYEB with 0.1 mM brassinin, pre-grown mycelia of the wild type digested about 50% and 100% of the brassinin respectively during 4 and 8 h of incubation (Figure 1D). The mutant mycelia, however, did not degrade a measurable amount of brassinin during 8 h of growth and over 80% of the brassinin still remained after 24 h. We investigated the effects of brassinin on gene expression in nutrient-rich GYEB after actively growing mycelium had been exposed to brassinin for 4 h. This specific time was selected because the amount of brassinin was reduced by 50% in the medium with wild type but it was not reduced in the medium with Δ*bdtf1*mutants (Figure 1D).

### 2.2. Gene Expression during Plant Infection

We used green cabbage (*B. oleracea*) to compare gene expression profiles between the mutants and wild-type *A. brassicicola* during the infection of host plants. Infection protocol using green cabbage was well established in our laboratory and most previous experiments were performed with this plant [11,13]. In addition, significant differences in the diameters of lesions on green cabbage between the wild type and mutants became noticeable at about 44 hpi. We speculated that the smaller lesions caused by the mutants were due to the induction of brassinin by the plant around 44 hpi. Notably, four phytoalexins, including brassinin and its derivatives, were induced and detected 44 h post-inoculation (hpi) in *Brassica junceae* [2]. Thus, we also compared gene expression profiles during the infection of green cabbage at 44 hpi as a complementary experiment.

### 2.3. Statistics of Gene Expression Profiles

Infection samples of mixed tissue from host plants and fungal hyphae at 44 hpi produced a total of 90.2 and 66.8 million reads of sequence tags for the wild type and Δ*bdtf1* mutants, respectively. Of these, respective 5.42 × 10^7^ (60%) and 4.01 × 10^7^ (60%) were mapped to the genome of *A. brassicicola*. Among 10,688 predicted genes in the *A. brassicicola* genome [14], 93 genes in the Δ*bdtf1* mutant were expressed at levels over twofold lower and 170 genes were expressed at levels over twofold higher (*p* < 0.05) than the wild type (Table S1).

Fungal tissue samples from GYEB with 0.1 mM brassinin produced a total of 61.9 and 77.2 million reads for the wild type and Δ*bdtf1* mutant, respectively. Of these, 4.60 × 10^7^ (74%) and 6.83 × 10^7^ (88%) were mapped to the genome of *A. brassicicola*. Among 10,688 predicted genes in the *A. brassicicola* genome, 560 genes from the ∆*bdtf1* mutants were expressed at levels over twofold lower and 388 genes expressed at levels over twofold higher (*p* < 0.05) than the wild type (Table S2). No sequence tags of the *Bdtf1* gene were expressed by the ∆*bdtf1* mutants in either set of data (Table S1 and S2). We examined the reliability of the RNA-seq data with semi-quantitative real time polymerase chain reaction (qRT-PCR) using four differentially expressed genes: *Bdtf1*, BHAb, AB02263.1, and AB08641.1. The qRT-PCR results were similar to the RNA-seq data (Tables S1 and S2).

### 2.4. Brassinin Effects: Genes at Lower Levels in the Mutant

During mycelial growth the Δ*bdtf1* mutant expressed fewer transcripts of 560 genes than the wild type in the presence of brassinin. Among the 560 genes, 286 had no homologs with functional annotations in public databases and the other 274 had similar genes either in predicted protein sequences or well-studied functional domains. The 274 genes belonged to the categories of helicases, nucleotide binding proteins, DNA-dependent ATPases, and ATP binding proteins (Table 1). Many of these genes were associated with ribosome biogenesis. These genes included eight RNA-binding proteins, three RNA polymerases, three ribosomal proteins, two ribonucleases, pre-rRNA processing complex protein, rRNA processing proteins, and 20 helicases. Other genes included several proteins associated with tRNA processing and translation initiation (Table S2). In contrast, there were few or no genes directly associated with DNA replication, transcription, recombination, or DNA damage-repair. The expression of HSP70 (AB02816.1) was reduced more than twofold (*p* < 0.05) in the mutant during mycelial growth in the presence of brassinin but was not affected during plant infection. The expression level of HSP90, which is activated by brassinin [15], was similar between the mutant and wild type. Expression levels of *AbSlt2* and *AbHog1* activated by brassinin [6], possibly via phosphorylation, were also similar between the wild type and the mutant.

**Table 1:** Functional groups of proteins over-represented among 560 genes that were expressed at lower levels in the mutant compared to wild-type *Alternaria brassicicola* during saprophytic growth in the presence of brassinin.

Annotation	Description	*p*-value
GO:0043141	ATP-dependent 5'-3' DNA helicase activity	0.000191
GO:0008026	ATP-dependent helicase activity	0.000191
GO:0008758	UDP-2,3-diacylglucosamine hydrolase activity	0.000191
GO:0047429	nucleoside-triphosphate diphosphatase activity	0.000191
GO:0004787	thiamin-pyrophosphatase activity	0.000191
GO:0008413	8-oxo-7,8-dihydroguanine triphosphatase activity	0.000191
GO:0004386	helicase activity	0.000191
GO:0019176	dihydroneopterin monophosphate phosphatase activity	0.000191
GO:0019177	dihydroneopterin triphosphate pyrophosphohydrolase activity	0.000191
GO:0008828	dATP pyrophosphohydrolase activity	0.000191
GO:0000810	diacylglycerol pyrophosphate phosphatase activity	0.000191
GO:0043139	5'-3' DNA helicase activity	0.000191
GO:0005488	binding	0.000213
GO:0030554	adenyl nucleotide binding	0.000213
GO:0017110	nucleoside-diphosphatase activity	0.000213
GO:0008796	bis(5'-nucleosyl)-tetraphosphatase activity	0.000312
GO:0004551	nucleotide diphosphatase activity	0.000312
GO:0000166	nucleotide binding	0.000587
GO:0003678	DNA helicase activity	0.000629
GO:0004003	ATP-dependent DNA helicase activity	0.000629
GO:0003676	nucleic acid binding	0.000772
GO:0017076	purine nucleotide binding	0.001009
GO:0008094	DNA-dependent ATPase activity	0.001162
GO:0003824	catalytic activity	0.002675
GO:0017171	serine hydrolase activity	0.004091
GO:0004086	carbamoyl-phosphate synthase activity	0.004091
GO:0008236	serine-type peptidase activity	0.004091
GO:0016887	ATPase activity	0.01031
GO:0005730	nucleolus	0.010587
GO:0050660	FAD binding	0.010587
GO:0048037	cofactor binding	0.012798
GO:0032040	small subunit processome	0.013819
GO:0031177	phosphopantetheine binding	0.020373
GO:0017111	nucleoside-triphosphatase activity	0.029526
GO:0016638	oxidoreductase activity, acting on the CH-NH2 group of donors	0.031008
GO:0005524	ATP binding	0.037275
GO:0032559	adenyl ribonucleotide binding	0.037275
GO:0016874	ligase activity	0.042934
GO:0006537	glutamate biosynthetic process	0.043581
GO:0042623	ATPase activity, coupled	0.049164

### 2.5. Brassinin Effects: Genes at Higher Levels in the Mutant

During mycelial growth in the presence of brassinin, the mutant expressed more transcripts of 388 genes than the wild type. Of the 388 differentially expressed genes, 238 did not have similar genes in the public databases. The other 150 genes had similar sequences with functional annotations. Representative molecular functions of these 150 genes included catalytic activity, biological processes, metabolism, and hyrolase activity. Genes associated with cellular processes (GO:0009987) or cellular metabolic processes (GO:0044207) were less common (*p* < 0.05) among the differentially expressed genes than those randomly selected in the genome. In contrast, drug-resistance proteins and ABC transporters were over-represented (Table 2). Lipid metabolic process (GO:0006629), membrane transport (GO:0016021) and oxidative stress response (GO:0006979) genes were among the genes expressed in greater amounts by the mutants than by the wild type.

**Table 2:** Functional groups of proteins under- or over-represented among 388 genes that were expressed at higher levels in the mutant compared to wild-type *Alternaria brassicicola* during saprophytic growth in the presence of brassinin.

Annotation	Description	Representation	*p*-value
GO:0009987	cellular process	Under	0.001924
GO:0044237	cellular metabolic process	Under	0.013208
KOG0065	Pleiotropic drug resistance proteins (PDR1-15), ABC superfamily	Over	0.041251
PF06422	CDR ABC transporter	Over	0.044766

### 2.6. Gene Expression Patterns During Plant Infection

There were 93 genes expressed at lower levels in the mutant than in the wild type during plant infection. These genes included a few putative hydrolytic enzyme-coding genes, such as alcohol dehydrogenases, isochorismatase hydrolases, and mannose dehydrogenase. None of these genes formed a coherent functional group that was over-represented with statistical significance. However, AB02597.1, AB03046.1, AB07427.1, and AB10411.1 of 90 Na+/Pi symporter (KOG2493) were expressed at lower levels in the mutant than the wild type. Interestingly, the 170 genes that were expressed at twofold higher levels in the mutant compared to the wild type included many genes with hydrolyase activity (Table 3). These genes encode putative cell-wall degrading enzymes, such as a cutinase (AB1674.1), five pectate lyases (AB0565.1, AB0904.1, AB01332.1, AB04736.1, and AB04813.1) and 22 glycoside hydrolases (Table S1).

**Table 3:** Functional groups of proteins over-represented among 170 genes that were expressed at higher levels in the mutant than in wild-type *Alternaria brassicicola* during infection of the host plant, *Brassica oleracea*.

Annotation	Description	*p*-value
GO:0016798	hydrolase activity acting on glycosyl bonds	7.38 × 10^−9^
GO:0004553	hydrolase activity, hydrolyzing O-glycosyl compounds	7.38 × 10^−9^
GO:0005975	carbohydrate metabolic process	5.80 × 10^−8^
GO:0005622	intracellular	9.67 × 10^−4^
GO:0044424	intracellular part	0.025823
GO:0006139	nucleobase, nucleoside, nucleotide and nucleic acid metabolic process	0.025823

### 2.7. Fungal Genes Affected Under Both Conditions

**Figure 2:**
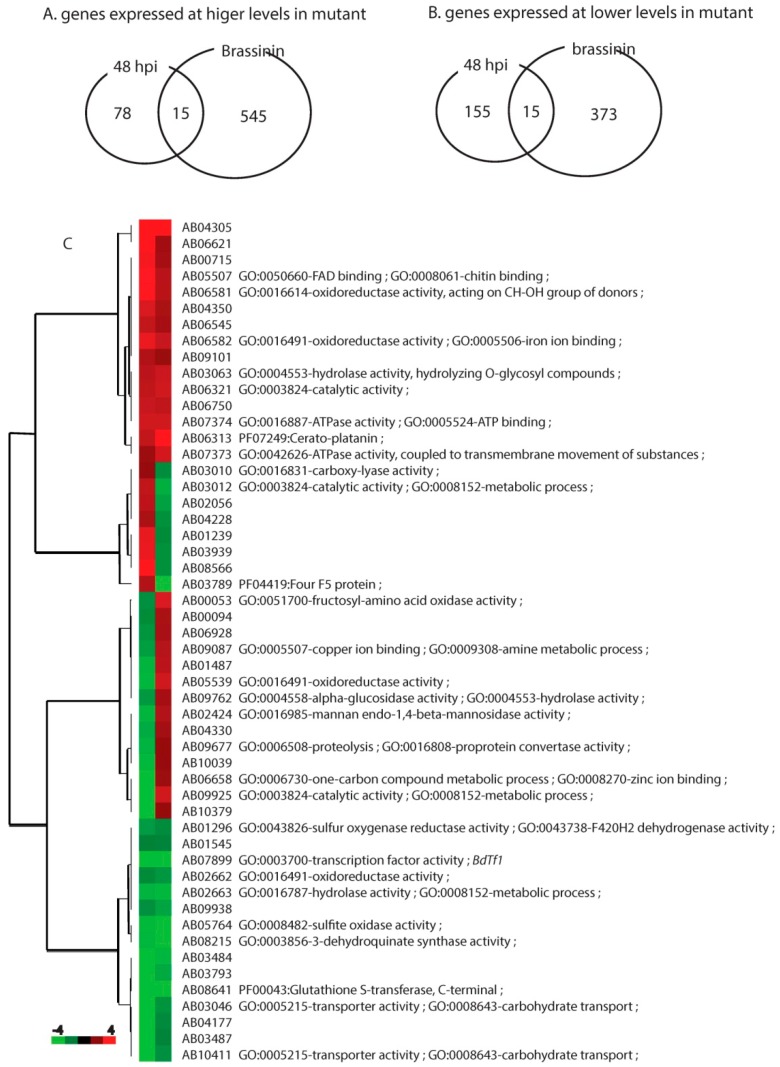
Hierarchical clustering of fungal RNA-seq data showing the number of overlapping genes among four groups of differentially expressed genes. (**A**) Number of genes expressed at higher levels in the mutant than in the wild type during host infection (44 hpi) and mycelial growth in the presence of brassinin. (**B**) Number of genes expressed at lower levels in the mutant than in the wild type. (**C**) Set of 52 genes showing differential expression patterns between the mutant and the wild type. The color key represents the log2 ratio of fragments per kilobase of exon model per million. Red indicates higher expression levels and green indicates lower expression levels in the mutant than in wild-type *A. brassicicola*. Abbreviations: number of genes differentially expressed in the mutant during mycelial culture in the presence of 0.1 mM brassinin; 44 hpi = number of genes differentially expressed in the mutant during plant infection

Among several hundred genes affected by the *Bdtf1* loss-of-function mutation, we identified 52 genes that were differentially expressed under both experimental conditions (Table S3). Thirty of them were expressed consistently at either higher or lower levels in the mutant than in the wild type. We speculated that the brassinin digestion enzymes were induced in the presence of brassinin and that the *Bdtf1* gene was essential for the induction of those genes. Furthermore, the mutant was less virulent mainly due to its inability to detoxify brassinin. Thus, we were interested in genes that were expressed at lower levels in the mutant than the wild type under both experimental conditions. There were 15 such genes among the 52 differentially expressed genes (Figure 2). As suspected, the *Bdtf1* gene was not expressed in the mutant under either condition but was expressed in wild-type *A. brassicicola* (Table 4). One gene (AB08641.1) showed a 41-fold difference in its expression during mycelial growth in the presence of brassinin. The ratio was over 10-fold during plant infection. The predicted amino acid sequence of the gene showed a low sequence similarity to glutathione S-transferease. Except for the *Bdtf1* and AB08641.1 genes, expression ratios between the wild type and mutant were modest for the other 13 of 15 genes. These 13 remaining genes encoded diverse enzymes and 2 transporters (Figure 2).

## 3. Discussion

### 3.1. Effects of Brassinin on Protein Synthesis

Wild-type *A. brassicicola* promptly detoxified brassinin, thus overcoming the inhibition effects on mycelial growth, unlike *∆bdtf1* mutant (Figure 1). Over 40 of the 274 genes with annotation that were expressed at lower levels in the mutant than in the wild type were putatively associated with protein synthesis (Table S2). We speculate that the reduced expression of these 40 genes was caused by the brassinin rather than by deletion of the *Bdtf1* gene. It is possible that cellular metabolism was significantly slowed in the mutant during mycelial growth *in vitro* with brassinin and transcription and translation were slowed accordingly.

It is notable that camalexin appears to inhibit protein synthesis [16]. We propose a possibility that brassinin also negatively affect protein synthesis in a concentration-dependent manner. The concentration of brassinin in the culture medium of the mutant was higher than the medium of the wild type after 4 h incubation although equal amounts were initially added to the medium. This higher concentration of brassinin might have caused a strong suppression of these 40 genes associated with protein synthesis. The possibility that both camalexin and brassinin inhibit protein synthesis warrants further investigation.

**Table 4:** Genes expressed at lower levels in the mutant compared to wild-type *Alternaria brassicicola* during saprophytic growth in the presence of brassinin and during the infection of host plants.

ProteinID	HMM-Secretion	^2^ Mycelial Growth with Brassinin		^3^ Plant Infection		Go Annotation	Manual Blast Results
		Wild type	∆*bdtf1*	Wild type	∆*bdtf1*		
AB01296.1		10.3	4.5	3.1	1.4	GO:0043826-sulfur oxygenase reductase activity;	Aldo/keto reductase are major group of enzymes involved in detoxification
AB01545.1		199.8	99.0	195.2	95.9	NA	xanthine phosphoribosyltransferase or purine salvage enzyme
AB02662.1		199.2	93.9	95.3	41.9	GO:0016491-oxidoreductase activity;	Aldo/keto reductase involved in detoxification
AB02663.1	^1^ S	100.7	37.0	33.0	10.4	GO:0016787-hydrolase activity ;	HAD-superfamily subfamily IIA hydrolase
AB03046.1		94.4	17.6	177.8	80.5	GO:0005215-transporter activity;	Sugar transporter STL1 induced when cells are subjected to osmotic shock
AB03484.1		75.9	13.3	75.6	25.6	NA	Similar to glutathione-dependent formaldehyde-activating GFA
AB03487.1		3.9	0.7	20.9	10.3	NA	Glutathione S-transferase omega-like
AB03793.1		4.6	1.1	54.3	21.8	NA	Cupin domain, salicylate hydroxylase
AB04177.1		5.3	1.1	73.6	35.1	NA	Methyltransferase involved in epigenetic regulation.
AB05764.1		3.0	0.9	1.4	0.3	GO:0008482-sulfite oxidase activity;	nitrate reductase
AB07899.1		11.0	0.0	29.7	0.1	GO:0003700-transcription factor activity;	*Bdtf1*
AB08215.1		5.1	1.7	56.7	8.5	GO:0003856-3-dehydroquinate synthase activity;	3-dehydroquinate synthase
AB08641.1		18.7	0.4	185.1	15.2	NA	glutathione S-transferase
AB09938.1		129.7	59.7	61.0	24.8	NA	arginine N-methyltransferase
AB10411.1		72.5	12.4	110.7	50.9	GO:0005215-transporter activity;	Sugar transporter STL1

^1.^ “S” indicates a putative secretion protein predicted by hidden Markov models and signal P. ^2.^ Numbers in these columns indicate normalized expression levels of each gene represented by fragments per kilobase of exon model per million (FPKM). ^3.^ Numbers in these columns indicate normalized expression levels of each gene represented by FPKM during host plant infection.

### 3.2. Genes Important for Cell Protection

Brassinin affected germination and mycelial growth of the mutant strains (*∆bdtf1*) more than it affected wild-type *A. brassicicola*. Neither was killed by the brassinin, however, even when exposed for several days to the concentration that totally inhibited germination and mycelial growth of the mutant [11]. The phytoalexin camalexin elicits genes involved in the biosynthesis of sterol, sphingolipid, and melanin [5]. These three compounds are probably important for cell protection against oxidative damage. Likewise, *∆bdtf1* mutants unable to detoxify brassinin may induce other genes to mitigate its effects during exposure. We inquired whether *Bdtf1* was important for the maintenance of cell wall integrity by comparing the gene expression profiles between the wild type and the *∆bdtf1* mutant. Differentially expressed genes did not include those associated with biosynthesis of sterol, sphingolipid, or melanin. It is possible that the expression level of these genes was elevated in both the wild type and mutant when exposed to brassinin. In addition, the expression level of the *AbHog1* or *AbSlt2* genes of *A. brassicicola* was similar in the wild type and the mutant after exposure to the brassinin. Loss-of-function mutants of either gene are hypersensitive to brassinin [6]. The *∆bdtf1* mutant was also hypersensitive to brassinin, but there was no significant difference in the expression levels of *AbHog1* and *AbSlt2* genes in the *∆bdtf1* mutant or wild-type *A. brassicicola*. Furthermore, the *∆bdtf1* mutant did not have defects in osmoregulation, cell wall integrity, or oxygen stress response during mycelial growth in the absence of brassinin [11]. The gene-expression pattern and the cell-wall related phenotypes of the mutant suggest that the *Bdtf1* gene is not a downstream gene of either the *AbHog1* or *AbSlt2* gene. It also suggests that the *Bdtf1* gene is not important for the maintenance of cell wall integrity or membrane biogenesis.

### 3.3. Compensatory Genes

Another phytoalexin, camalexin, induces drug-efflux genes in *A. brassicicola* [5] and activates brassinin-detoxifying enzymes in mycelial cultures of *A. brassicicola* [2]. It is of note that the *∆bdtf1* mutant was not killed in the presence of brassinin [11] and the brassinin was reduced by 20% after 24 h of incubation (Figure 1D). Survival of the mutant might have been possible by the genes expressed at higher levels in the mutants than in the wild type in response to brassinin. They included 12 and 15 genes encoding transporters and putative detoxifying enzymes, respectively. Expression of one of the transporter-coding genes (AB04925.1), for example, was five times greater in the mutant. It is possible that this higher level of expression was caused by the greater concentration of brassinin and was involved in limiting the intracellular accumulation of brassinin by pumping it out. Putative toxin digestion enzymes included three oxidoreductases, three carboxylesterases, two cyanide hydratases, two heat shock proteins, cyanate lyase, amidase, hydroperoxide reductase, latamase, monooxygenase, oxidase, multicoper oxidase, and peroxidase. Some of these genes might have been responsible for the slight reduction of brassinin.

### 3.4. Enzymatic Modification of Brassinin

The ability of plant-pathogenic fungi to promptly detoxify plant defense compounds is an important determinant of their virulence [17,18]. Brassinin hydrolase (BHAb), a detoxifying enzyme in *A. brassicicola* [12], however, was not included in the list of differentially expressed genes. This result indicates that the *Bdtf1* gene does not regulate *BHAb*, as we found in a previous study [11]. We doubt that other genes encoding putative enzymes described above as compensatory genes are the brassinin digestion enzymes regulated by the *Bdtf1* gene. If they were regulated by *Bdtf1* gene, their expression should have been reduced in the *∆bdtf1* mutant. However, they were expressed at a higher level and the mutant was still unable to detoxify brassinin. Thus, unknown brassinin detoxifying enzymes among those expressed at lower levels in the mutant than the wild type in the presence of brassinin are yet to be discovered.

### 3.5. Candidates for Brassinin-Detoxifying Enzymes

The brassinin derivatives brassilexin and cyclobrassinin have stronger antifungal activities than brassinin [19]. Wild-type *A. brassicicola* must detoxify brassinin for successful pathogenesis. It is unclear whether the fungus also processes other brassinin-derived phytoalexins or exhaustively digests brassinin before it is converted to its derivatives. Either way, comparisons of the gene expression profiles between wild-type *A. brassicicola* and the mutant ∆*bdtf1* provided data leading to the discovery of enzymes involved in the detoxification of brassinin. Brassinin is detoxified through oxidation or hydrolysis as implied by chemical analysis [2,20]. The 15 genes expressed at lower levels in the mutant included a hydrolase (AB02663.1), two GST-like proteins (Ab08641.1 and AB03487.1), and three reductases (Table 4). Expression ratios were over fivefold lower in the mutant than the wild type for two GST-like protein genes. Other genes showed small differences. It is yet to be verified experimentally if any of these genes encode enzymes that detoxify brassinin. A loss-of-function mutation of the genes that detoxify brassinin would reduce virulence in the mutant compared to wild-type *A. brassicicola*. The severity of the reduction would depend on several factors. First, functional redundancy among the detoxifying enzyme genes will influence the effects of the loss-of-function mutation of each gene. A lack of redundancy would cause the greatest reduction in virulence. Second, an enzyme cascade would also affect virulence. Intermediate products produced by one enzyme can accumulate and slow further conversion of brassinin. The brassinin derivatives make the outcome even more complicated. Loss-of-function mutants of each gene need to be developed and their effects on virulence clarified. The enzyme activities of the proteins encoded by each gene also need to be verified.

## 4. Experimental Section

### 4.1. Fungal Strains and their Maintenance

We used the facultative plant pathogen *Alternaria brassicicola* (Schweinitz, Wiltshire, UK) (ATCC96836) in this study. Fungal strains of the wild type and its mutants, *∆bdtf1-5* and *∆bdtf1-9*, were purified by two rounds of single-spore isolation. To restore their vigor, each strain was inoculated on host plants and the conidia produced were transferred to potato dextrose agar. Newly formed conidia were harvested from the agar after 5 days of growth. The conidia were suspended in 20% glycerol and maintained as culture stock in separate tubes, with one tube used for each assay as described previously [13,21].

### 4.2. Assays for Brassinin Digestion and Preparation of Mycelium for RNA-Seq

Fungal mycelia were grown for 2 days in 1% glucose and 0.5% yeast extract broth (GYEB). The medium was refreshed 16 h before harvest. Mycelia were harvested and semi-dried by blotting with sterile paper. Subsequently, 0.15 g of semi-dried mycelium (equivalent to 0.025 g dry weight) was transferred to GYEB containing 0.1 mM brassinin. The mycelia were cultured at 25 °C in a shaker-incubator for 24 h with continuous agitation at 100 rpm. During the 24 h, 2 mL of GYEB was recovered from each culture flask at 4, 8, and 24 h. After removal of the mycelia, GYEB from each culture was transferred to a clean tube and extracted twice using 0.8 mL of chloroform for each extraction. The relative concentration and integrity of the brassinin were evaluated using a HPLC system as previously described [11]. This experiment, from the growth of fungal strains to brassinin quantification, was performed three times. For gene expression profiles, mycelia were harvested 4 h after the transfer to brassinin-containing medium. They were semi-dried by blotting with paper towels, immediately frozen by plunging them into liquid nitrogen, and then stored at −70 °C. A total of three sets of tissue were separately prepared as three biological replicates.

### 4.3. Preparation of Fungal Tissues from Infected Host Plants

We performed pathogenicity assays as described previously, with a slight modification [11,22]. Two healthy leaves were harvested from each of nine host plants of *B. oleracea* and placed in mini-moist chambers. Each leaf was inoculated with six droplets of wild-type inoculum on the left side and six droplets of a *∆bdtf1* mutant strain on the right side of the central vein. The inoculum contained 1,500 conidia of either the *∆bdtf1* mutant or wild-type *A. brassicicola* in 10 µL of water. The mini-moist chambers were sealed with plastic wrap after leaf inoculation to keep the relative humidity close to 100%. Host plant tissue and fungal hyphae were harvested at 44 hpi from six leaves (three plants) for each sample. The tissues were frozen in liquid nitrogen immediately after harvest to fix gene expression profiles. Three sets of tissues were harvested for each strain as three replicates.

### 4.4. Generation of RNA-Seq Data

We extracted total RNA from the frozen tissues using an RNeasy kit and residual DNA was digested in columns following the manufacturer’s protocol (Qiagen, Palo Alto, CA, USA). With the DNA-free RNA, we constructed strand-specific sequencing libraries using the TruSeq™ RNA Sample Prep Kit following the manufacturer’s protocol (Illumina, San Diego, CA, USA). Each library representing a replicate sample was constructed with a unique index primer. A total of six index primers were used to construct six libraries. All six libraries were mixed and 100 nucleotide-long sequence tags were determined using Illumina Hiseq2000 (Illumina). Image analysis, base-calling, and quality checks were performed with the Illumina data analysis pipeline CASAVA v1.8.0. The data were deposited in NCBI’s Gene Expression Omnibus and are accessible through GEO Series Accession No. GSE59195.

The sequenced reads were mapped to the genome sequence of *A. brassicicola* in the interactive JGI fungal portal MycoCosm [23,24] using the programs Tophat 2.0.0 [25] and Bowtie 2.0.0 [26]. Default settings were used, except in the case of *A. brassicicola* the intron length was designated as a minimum of 10 nucleotides and a maximum of 500 nucleotides. The program Cuffdiff version 1.3.0, part of Cufflinks [27], was used to identify reads overlapping with previously predicted genes. The mapping bias correction method was used while running Cuffdiff [28]. The expression levels of each predicted gene were determined and normalized to Mapped Fragments Per Kilobase of exon model per Million (FPKM). Differentially expressed genes between the wild type and the mutant were determined by comparing FPKM from three biological replicates for both the wild type and the mutant using the default-allowed false discovery rate (FDR) of 0.05. In addition to this we applied a cutoff of at least a twofold change in expression value for differential expression. Custom scripts were written in Python for data analysis.

#### Representation Analysis of Functional Annotation Terms

Custom scripts were developed in Python and R to analyze over- and under-representation of functional annotation terms in sets of differentially regulated genes using the Fisher Exact test. The Benjamini-Hochberg correction was used to correct for multiple testing using a *p*-value of 0.05.

## 4.5. qRT-PCR

We generated a cDNA pool using Superscript II from 2 µg of total RNA for each sample following manufacturer’s protocol (Invitrogen, Carlsbad, CA, USA). Subsequent semi-quantitative PCR was performed as described previously [22]. Relative amounts of transcripts for each gene were calculated compared to the housekeeping gene, elongation factor 1-α (*Ef1-α*) by [(number of transcripts of a gene) / (number of transcripts of *Ef1-α*)] × 100. The *Ef1-α* gene showed consistent expression patterns in all tissue samples studied previously [22,29,30]. Thus, we used it as a representing house-keeping gene to calculate relative expression levels of four genes, including the transcription factor *Bdtf1* (AHU86567.1). Brassinin hydrolase in *A. brassicicola* (BHAb, AB00197.1) was included to confirm previous study results [11,12]. We also included two genes (AB02663.1, AB08641.1) that showed differential expression under both experimental conditions. Pairs of primers were designed and used for the qRT-PCR with *BdTf1*, *BHAb*, AB02663.1, AB08641.1 and *Ef1-α* genes. Primers are Bdtf1rtF (GTCAGAGCATAGCCGACACA) Bdtf1rtR (TGAAGCTTCGGAGGAAAGAG), BHAbrtF (TTCT GGTGGAGAGGGAGCTA), BHAbrtR (GGATCCTGATAGAGCCACCA), AB02663RtF (CCCGAA CTGGCTACCTACAA) AB02663RtR (GAAGCAGGGTTGTCACCAAT), AB8641RtF (AACCCCA AAGGCAGAATACC) AB8641RtR (ATTTCTTTTCGGGGACGAGT) and Ef1αrtF (GGGTCCTC GACAAGTTGAA), and Ef1αrtR (GGGAGCGTCAATAACTGTGA).

## 5. Conclusions

Brassinin is detoxified through oxidation or hydrolysis as implied by chemical analysis and the enzymes responsible for the modification are yet to be identified. Previously we identified a transcription factor that is essential for efficient detoxification of brassinin *in vitro*. To discover enzyme-coding genes involved in the brassinin detoxification, we compared gene expression profiles between wild-type *A. brassicicola* and the mutant ∆*bdtf1* under two different experimental conditions. In this study, we discovered six candidate genes, including a hydrolase (AB02663.1), two GST-like proteins (AB08641.1 and AB03487.1), and three reductases. We have generated loss-of-function mutants of genes encoding either a hydrolase or one of the two GST-like proteins. We are in the process of verifying their functions in brassinin detoxification *in vitro*.

## Supplementary Materials

Supplementary materials can be accessed at: http://www.mdpi.com/1420-3049/19/8/10717/s1.

## Supplementary Files

Supplementary File 1
